# A Nationwide Study of Norwegian Patients with Hereditary Angioedema with C1 Inhibitor Deficiency Identified Six Novel Mutations in *SERPING1*

**DOI:** 10.1371/journal.pone.0131637

**Published:** 2015-07-08

**Authors:** Irene Johnsrud, Mari Ann Kulseth, Olaug Kristin Rødningen, Linn Landrø, Per Helsing, Erik Waage Nielsen, Ketil Heimdal

**Affiliations:** 1 Department of Dermatology, Oslo University Hospital, Rikshospitalet, N 0027, Oslo, Norway; 2 Department of Medical Genetics, Oslo University Hospital, N 0027, Oslo, Norway; 3 Department of Anesthesiology, Nordland Hospital, N 8092, Bodø, Norway; University of Thessaly, Faculty of Medicine, GREECE

## Abstract

Hereditary angioedema with C1 inhibitor deficiency (C1-INH-HAE) is characterized by relapsing, non-pruritic swelling in skin and submucosal tissue. Symptoms can appear in early infancy when diagnosis is more difficult. In the absence of a correct diagnosis, treatment of abdominal attacks often lead to unnecessary surgery, and laryngeal edema can cause asphyxiation. A cohort study of 52 patients from 25 unrelated families in Norway was studied. Diagnosis of C1-INH-HAE was based on international consensus criteria including low functional and/or antigenic C1-INH values and antigenic C4. As *SERPING1* mutations in Norwegian patients with C1-INH-HAE are largely undescribed and could help in diagnosis, we aimed to find and describe these mutations. Mutation analysis of the *SERPING1* gene was performed by Sanger sequencing of all protein coding exons and exon-intron boundaries. Samples without detected mutation were further analyzed by multiplex ligation-dependent probe amplification to detect deletions and duplications. Novel mutations suspected to lead to splice defects were analyzed on the mRNA level. Fifty-two patients from 25 families were included. Forty-four (84,6%) suffered from C1-INH-HAE type I and eight (15,4%) suffered from C1-INH-HAE type II. Pathogenic or likely pathogenic mutations were found in 22/25 families (88%). Thirteen unique mutations were detected, including six previously undescribed. There were three missense mutations including one mutation affecting the reactive center loop at codon 466, three nonsense mutations, three small deletions/duplications, three gross deletions, and one splice mutation.

## Introduction

Hereditary angioedema with C1 inhibitor deficiency (C1-INH-HAE) is a rare autosomal dominant disease with an uncertain prevalence estimated to be 1: 50 000 [1] and is caused by mutation in the *SERPING1* gene. It is characterized by non-pruritic, episodic and self-limiting swellings in skin and submucosal tissue without urticaria. Laryngeal edema can be fatal. Two variants of C1-INH-HAE exist, C1-INH-HAE type I and type II. Hereditary angioedema with normal C1-inhibitor (nC1-INH HAE) is believed not to be caused by *SERPING1* mutations. The condition is more likely due to mutations in coagulation factor XII or other unknown causes. These patients show normal values for C4, C1-INH level and C1-INH function and are not included in our study.

The expected 50% amount of C1 inhibitor (C1-INH) secreted from the normal allele in heterozygous C1-INH-HAE patients is rapidly consumed and usually only 10% is measurable in plasma. Both C1-INH-HAE type I and type II have decreased C1-INH activity. C1-INH-HAE type I has low amount of plasma C1-INH caused by lack of production from the mutated allele or production of a protein unrecognized in the antigen analysis. C1-INH-HAE type II has low amount of functional C1-INH in plasma and usually increased amounts of abnormal C1-INH. The mutated allele produces a C1-INH protein unable to make complexes with target proteases. This defect C1-INH protein is detected by antigenic tests and is slowly cleared from circulation. With very few exceptions both C1-INH-HAE type I and II patients show reduced concentrations of the complement component C4, even during asymptomatic periods [2].

C1-INH inhibits the enzymes C1r, C1s, mannose-associated serine protease (MASP) -1 and -2 in the complement system, activated factor XII, clotting factor XI, plasma kallikrein, plasmin and factor seven activating protease (FSAP) [3–5]. A deficiency of C1-INH results in an uncontrolled activation of these cascade systems. Uncontrolled activation of kallikrein increases the cleavage of high molecular weight kininogen (HMWK) and releases bradykinin. Bradykinin is responsible for plasma leakage and edema. Histamine and mast cells are not directly involved, and C1-INH-HAE-patients do not respond to treatment with steroids and/or antihistamines.

C1-INH is encoded by *SERPING1* and C1-INH-HAE is caused by mutations in the *SERPING1* gene located at 11q12.1. The gene consists of 8 exons, of which 7 are protein coding (NM_000062.2). The intronic regions present a high density of interspersed repetitive Alu-elements, which makes the gene prone to deletions and duplications which are responsible for approximately 15% of all C1-INH-HAE cases [6]. To date, more than 450 different mutations throughout the whole gene have been reported (HGMD database, accessed Mai 2015) [7]. As mutations are *de novo* in approximately 25% of cases [8], patients may lack positive family history.

C1-INH is a member of the serpin-family. It is a glycoprotein of 478 aminoacids [9]. Five hundred amino acids are originally translated, but a signal peptide of 22 amino acids is split off before C1-INH reaches the circulation. The reactive centre loop of C1-INH in the carboxy terminal is recognized by target proteases which cleave C1-INH and bind irreversibly to the Arg 466-Thr467 (previously referred to as Arg 444-Thr 447). The cleavage induces a conformational change in C1-INH and the proteases permanently lose their enzymatic activity. C1-INH can also be cleaved by proteases without forming a covalent complex. If so, C1-INH can still form non-covalent interactions with e.g. bacteria, other proteins, cell surfaces and lipids via the heavily glycosylated amino-terminal end [10]. The composition of the amino-terminal domain has no functional impact, but is important for the stability of C1-INH in plasma [11].

Early diagnosis of C1-INH-HAE is vital since attacks can be treated efficiently and unnecessary examinations and surgery can be avoided. C1-INH-HAE might be difficult to diagnose in newborns and children [12]. In two cousins C1-INH values at birth and at 1–2 years were comparable. At 19 months of age one of the cousin had abdominal pain, distention and massive watery diarrhea. The symptoms persisted for 4 weeks, but subsided after 6 hours when 500 units of C1-INH concentrate was given. Later C1-INH-HAE diagnosis was confirmed in this cousin and ruled out in the other [12]. A genetic test is therefore of great value. The aim of this study was to identify mutations in the *SERPING1* gene in Norwegian C1-INH-HAE patients.

## Materials and Methods

### Patients

From September 4^th^ 2011 to Jan 1^st^ 2014, 52 patients from 25 families with the diagnosis C1-INH-HAE type I or type II were evaluated at the Department of Dermatology, Oslo University Hospital (OUS). C1-INH-HAE patients were referred by general practitioners or patients contacted the department directly for evaluation, treatment and genetic testing. Patients included were from most areas of Norway. The ages of the patients were from 18 and upwards. Diagnosis of C1-INH-HAE was established according to international consensus guidelines, based on clinical symptoms and serum levels of functional and antigenic C1-INH. Antigenic levels of C1-INH was measured using nephelometry (N Antisera to Human Coagulation Factors and C1 Inhibitor, BN II: Siemens Healthcare Diagnostics, Margburg, Germany) and C1-INH function with ELISA technique (Microvue complement/C1 Inhibitor Plus, Quidel, San Diego, USA), by the Department of Medical Immunology, Oslo University Hospital (OUS). Analyses were performed in accordance with the manufacturer`s instructions. Participating patients signed informed consent according to Norwegian regulations accepting gene mutation analysis. The study was approved by the regional committees for medical and health research ethics, South East Norway (2011/824).

### Molecular diagnosis

Genomic DNA was extracted from EDTA-anticoagulated whole blood using QIAsymphony DNA DSP Kit and QIAsymphony SP (Qiagen, Hilden, Germany) according to the manufacturer’s specifications.

Sequencing of the coding region (exons 2–8) and exon-intron boundaries (minimum +/- 20 nucleotides) of *SERPING1* (NM_000062.2) was performed using BigDye Terminator v.3.1 Cycle Sequencing Kit (Applied Biosystems, Foster City, USA) and analyzed on ABI 3730 DNA Analyzer (Applied Biosystems, Foster City, USA). Primer sequences and polymerase chain reaction (PCR) conditions are available on request. All coding exons were sequenced in both directions. Data were evaluated by using the sequence analysis and alignment software SeqScape (Version 2,5 Applied Biosystems, Foster City, USA).

### Multiplex ligand-dependent probe amplification (MLPA)

In order to search for large deletions or duplications, MLPA was performed using SALSA MLPA P243 *SERPING1* Kit (MRC Holland, The Netherlands) according to the manufacturer’s specifications. Data was analyzed with GeneMapper software v4.0 (Applied Biosystems, Foster City, California, USA).

### mRNA analyses of c.889+3A > T

A blood sample from the index patient was collected into PAXgene Blood RNA tubes and total RNA was extracted using the PAXgene Blood RNA kit (Qiagen, Valencia, CA, USA). RT-PCR was performed using Qiagen OneStep RT-PCR kit (Qiagen, Valencia, CA, USA) and primers located in exon 3 (5’-ACTCTCTGCTCTGACTTGGA-3’) and exon 6 (5’-CCACAGGGTACTTCTTGCTA-3’). The amplified products were analyzed on an Agilent 2000 BioAnalyzer (Agilent Technologies, Palo Alto, CA) and sequenced using the BigDye Terminator v3.1 Cycle Sequencing Kit (Applied Biosystems, Foster City, CA, USA). Data were evaluated by using the sequencing analyses software Sequencing Analyses 5.2 (Applied Biosystems, Foster City, California, USA).

Nomenclature of mutations follows the recommendations of the Human Genome Variation Society (http://www.hgvs.org/rec.html) for both DNA-level and protein-level. Thus, the first nucleotide (A) of the initiation methionine codon (ATG) of the cDNA sequence (GenBank accession number NM_000062.2) is nucleotide number one. For the protein amino acid positions, the 22 amino acids of the N-terminal residue of the signal peptide are included in the numbering. The exons are numbered systematically locating the initiating methionine codon (ATG) in exon 2.

## Results and Discussion

Mutation analysis in the *SERPING1* gene of patients with C1-INH-HAE have been performed in several countries over the recent years [13–15]. In this study we present the result of genetic analysis conducted on Norwegians patients with C1-INH-HAE type I and II. We have identified 52 patients from 25 families in Norway. In our cohort, two patients were from other European countries, one patient from South America and one from Central America. In this study, 16 families suffered from C1-INH-HAE type I and six families type II. 44 of 52 patients (84,6%) suffered from C1-INH-HAE type I, compatible with other European studies [14,16,17] and 8 of 52 patients (15,4%) suffered from C1-INH-HAE type II.

At ascertainment 18/25 (72%) index patients had a positive family history of C1-INH-HAE and 5/25 (20%) were new cases with negative family history.

We identified 13 mutations in *SERPING1*, where of six mutations are reported for the first time. We detected three missense mutations including one mutation affecting the reactive centre loop, Arg466, three nonsense mutations, three small deletions/duplications, three gross deletions, and one splice mutation. All novel mutations were identified in families with C1-INH-HAE type I. Five mutations were detected in more than one family (Table 1). A causative mutation was not identified in three families with C1-INH-HAE (type I). The finding of a causative mutation in 22/25 families gives a detection rate of 88%.

**Table 1:** SERPING1 variants identified by DNA sequencing or multiplex ligation-dependent probe amplification.

	cDNA numbering	Localization	Protein effect	Classification●	Type of mutation	C1-INH-HAE type	No. of families	Reference
1	c.19dupC	Exon 2	p.Leu7Profs*13	Likely pathogenic	Small dup	I	1	Novel
2	c.124G>T	Exon 3	p.Glu42*	Pathogenic	Nonsense	I	2	Pappalardo [16]
3	c.310C>T	Exon 3	p.Gln104*	Likely pathogenic	Nonsense	I	1	Gösswein [23]
4	c.360delG	Exon 3	p.Ser121Profs*27	Likely pathogenic	Small del	I	1	Novel
5	c.550G>A	Exon 3	p.Gly184Arg or splicing	Pathogenic	Missense/Splice defect	I	2	Kesim [24] Verpy [25] Bygum [13]
6	c.695T>G	Exon 5	p.Ile232Arg	Likely pathogenic	Missense	I	1	Novel
7	c.889+3A>T	Intron 5	p.Asp229_Ser296del	Likely pathogenic	Splice defect, skipping of exon 5	I	1	Novel
8	c.1232C>G	Exon 7	p.Ser411*	Pathogenic	Nonsense	I	2	Novel
9	c.1297delG	Exon 8	p.Asp433Thrfs*17	Likely pathogenic	Small del	I	1	Novel
10	c.1396C>T	Exon 8	p.Arg466Cys	Pathogenic	Missense	II	6	Skriver [18] Freiberger [26] Stein [27] Kalmar [28]
	**Deletions**							
11	del exon 1–4 (a)			Pathogenic	Gross deletion	I	1	Duponchel [29]
12	del exon 1–8 (b)			Pathogenic	Gross deletion	I	1	Duponchel [29]
13	del entire or part of exon 4			Pathogenic	Gross deletion	I	2	Duponchel [29] Gösswein [23] Verpy [20]

^(a)^ The upstream breakpoint for the deletion is located less than 364kb from exon 1. Downstream breakpoint is probably located within intron 4.

^(b)^ The upstream breakpoint for the deletion is located less than 364kb from exon 1. Downstream breakpoint is unknown.

^●^ Criteria for classification:

Likely pathogenic:

- nonsense mutations or out of frame indels.

- missense mutations previously reported in two families.

Pathogenic:

- nonsense mutations or out of frame indels previously reported.

- missense mutations previously reported in more than two families.

The distribution of mutation types in our population was unremarkable with three large deletions detected in 4/22 families and 10 mutations distributed throughout the entire gene (S1 Fig). All mutations were detected in heterozygous form. None of the identified variants are reported in control populations (Exome Aggregation Consortium (ExAC), Cambridge, MA (URL: http//exac.broadinstitute.org) 10^th^ of January 2015).

We were unable to detect a mutation in three families. Three affected members in a large mutation negative family were subjected to mRNA analysis. However, no appearance of missplicing was found and biallelic expression was detected. The utilized sequence analysis does not include regulatory regions and deep intronic sequences, and thus SERPING1 mutations in these families can not be excluded.

The most prevalent mutation detected in the Norwegian population was p.Arg466Cys which was detected in six families with C1-INH-HAE type II. To the best of our knowledge using genealogy and local knowledge of the origins of families, these are separate families. However, haplotyping has not been performed to exclude a founder situation. This strengthens previous observations of p.Arg466 as a hotspot for mutation and a major cause of C1-INH-HAE type II [18].

Four of the novel mutations introduce premature stop codons either directly (p.Ser411*) or by creating a frameshift via a single nucleotide duplication or deletion (p.Leu7Profs*13, p.Ser121Profs*27, p.Asp433Thrfs*17). Transcripts containing premature stop codons located more than 50 bp upstream of the last intron, are prone to degradation by nonsense mediated mRNA decay (NMD) leaving little or no transcripts for protein production. The single nucleotide deletion in exon 8 causes a frameshift encoding a truncated protein lacking the last 67 amino acids of C1-INH (p.Asp433Thrfs*17). The truncated protein lacks the important reactive central loop (Arg466 –Thr467) and is therefore likely to be dysfunctional.

One novel missense mutation c.695T>G, p.Ile232Arg was found in a Norwegian family. Xu et al.[15] detected another variant in the same position c.695T>A, p.Ile232Lys, in four Chinese C1-INH-HAE families. Both arginine and lysine are positively charged polar amino acids, and the effect of substituting the hydrophobic isoleucine with arginine or lysine is likely to be comparable. One novel splice site mutation, c.889+3A > T was detected. The variant is located in intron 5 and mRNA analysis performed on mRNA extracted from the index patients blood, revealed complete missplicing with deletion of exon 5 (S2 Fig). Deletion of exon 5 maintains the reading frame, encoding a protein p.Asp229_Ser296del which lacks the central part of the compact folded serpin domain of C1-INH [19] probably causing folding defect and endoplasmic reticulum-associated degradation. The previously reported c.550>A mutation, change the last nucleotide of exon 3 and is expected to modify the donor splice site of intron 3. The mutation may either cause aberrant splicing or an amino acid substitution (p.Gly162Arg) [13].

Two of the patients had deletions involving exon 4, and one patient had a deletion involving exons 1–4. Introns 3 and 4 contain several Alu repeats which might induce non-homologous recombination resulting in the observed deletions [4]. These variants were detected by MLPA and no further attempts to characterize the breakpoints were performed. Exon 4 deletions are in-frame and Verpy et al. [20] showed that deletion of exon 4 lead to formation of a protein which is retained in endoplasmic reticulum and not properly secreted.

The variant c.1232C>G is novel, however, Pappalardo [8] reported another substitution in the same position c.1232C>A. Both substitutions are expected to introduce a premature stop codon, p.Ser411*, creating a transcript prone to degradation by nonsense mediated mRNA decay.

This is the first study on the *SERPING1* gene in the Norwegian population. Pathogenic or likely pathogenic mutations were found in 22/25 families (88%). A genetic diagnosis might be helpful to reach a C1-INH-HAE diagnosis in difficult cases [1], and has been included in the laboratory diagnostic testing of C1-INH-HAE [21]. Genetic testing might be particularly useful in infants where protein analysis can be unreliable due to age-specific variability in C1-INH plasma concentration [22].

## Supporting Information

S1 Fig Distribution of disease causing mutations in *SERPING1* identified in the Norwegian families with hereditary angioedema with C1 inhibitor deficiency. (TIF)

S2 Fig a) SERPING1 mRNA exon 3 to 8. Location of primers used for RT-PCR, are indicated with arrows. The splice site variant (c.889+3A>T) and the polymorphism in exon 8 (c.1438G>A) are shown. b) RT-PCR with primers in exon 3 and 6 (black arrows). The electropherogram shows a mix of two transcripts, one normal and one that lacks exon 5. c) Specific amplification of normal transcript using primers in exon 5 and 8 (grey arrows). The electropherogram of the reverse sequence shows that the majority of the normal transcripts have a G (reverse sequence C) in position 1438, thus indicating monoallelic expression of normal transcript.
(TIF)
