# Correction: A Nationwide Study of Norwegian Patients with Hereditary Angioedema with C1 Inhibitor Deficiency Identified Six Novel Mutations in *SERPING1*

**DOI:** 10.1371/journal.pone.0136011

**Published:** 2015-08-13

**Authors:** Irene Johnsrud, Mari Ann Kulseth, Olaug Kristin Rødningen, Linn Landrø, Per Helsing, Erik Waage Nielsen, Ketil Heimdal

There are errors in the author affiliations. The affiliations should appear as shown here:

Irene Johnsrud^**1,2**^ Mari Ann Kulseth^**3**^, Olaug Kristin Rødningen^**3**^, Linn Landrø^**1**,**2**^, Per Helsing^**1**^, Erik Waage Nielsen ^**2,4,5,6**,^ Ketil Heimdal^**3**^

**1** Department of Dermatology, Oslo University Hospital, Rikshospitalet, N 0027 Oslo, Norway **2** Institute of Clinical Medicine, University of Oslo, N 0316 Oslo, Norway **3** Department of Medical Genetics, Oslo University Hospital, N 0027 Oslo, Norway **4** Department of Anesthesiology, Nordland Hospital, N 8092 Bodø, Norway **5** Faculty of Health Sciences, University of Tromsø, 9037 Tromsø, Norway **6** University of Nordland, Faculty of Professional Studies, 8049 Bodø, Norway

The order of S1 and S2 figs are switched. Please see the complete, correct S1 and S2 Figs here.

## Supporting Information

S1 Fig Distribution of disease causing mutations in *SERPING1* identified in the Norwegian families with hereditary angioedema with C1 inhibitor deficiency. (JPG)

S2 Fig a) *SERPING1* mRNA exon 3 to 8. Location of primers used for RT-PCR, are indicated with arrows. The splice site variant (c.889+3A>T) and the polymorphism in exon 8 (c.1438G>A) are shown. b) RT-PCR with primers in exon 3 and 6 (black arrows). The electropherogram shows a mix of two transcripts, one normal and one that lacks exon 5. c) Specific amplification of normal transcript using primers in exon 5 and 8 (grey arrows). The electropherogram of the reverse sequence shows that the majority of the normal transcripts have a G (reverse sequence C) in position 1438, thus indicating monoallelic expression of normal transcript.
(JPG)
