# Supplementary figures and images for: Correction: A Nationwide Study of Norwegian Patients with Hereditary Angioedema with C1 Inhibitor Deficiency Identified Six Novel Mutations in SERPING1
Source: PLoS One. 2015 Aug 13;10(8):e0136011. doi: 10.1371/journal.pone.0136011 (PMC4536201; doi:10.1371/journal.pone.0136011)

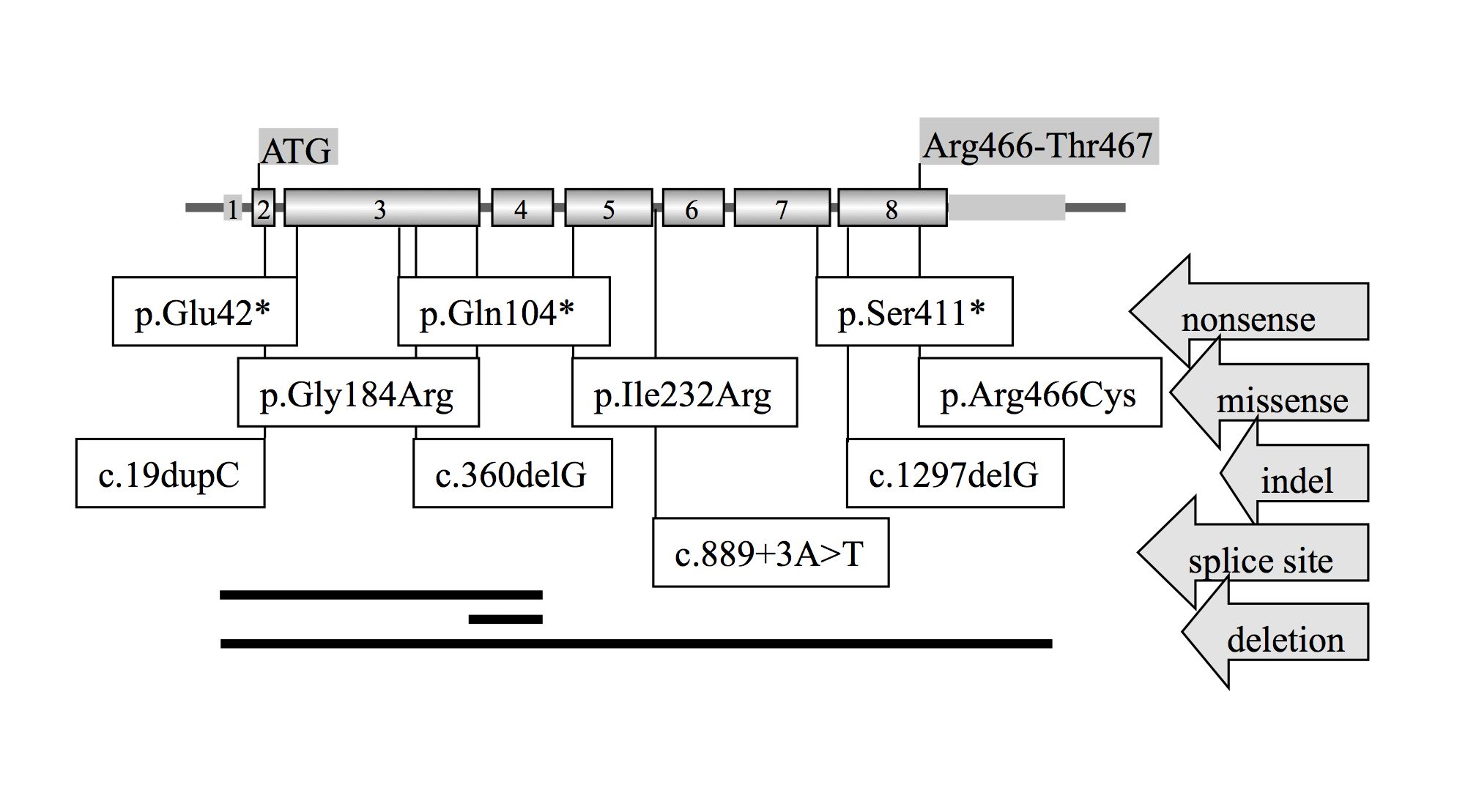

Supplement: S1 Fig — (JPG) [file pone.0136011.s001.jpg]

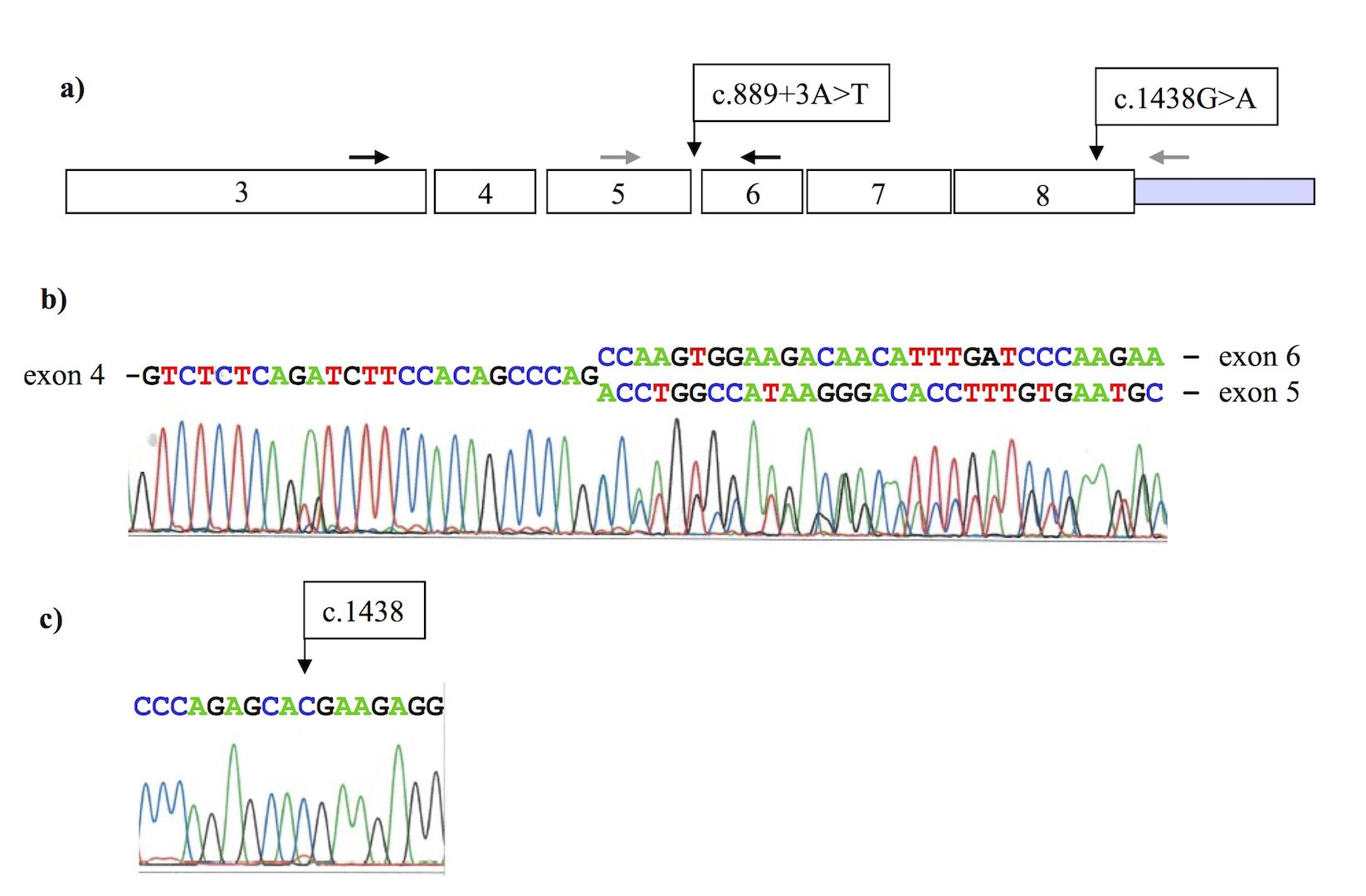

Supplement: S2 Fig — a) SERPING1 mRNA exon 3 to 8. Location of primers used for RT-PCR, are indicated with arrows. The splice site variant (c.889+3A>T) and the polymorphism in exon 8 (c.1438G>A) are shown. b) RT-PCR with primers in exon 3 and 6 (black arrows). The electropherogram shows a mix of two transcripts, one normal and one that lacks exon 5. c) Specific amplification of normal transcript using primers in exon 5 and 8 (grey arrows). The electropherogram of the reverse sequence shows that the majority of the normal transcripts have a G (reverse sequence C) in position 1438, thus indicating monoallelic expression of normal transcript. (JPG) [file pone.0136011.s002.jpg]
